# Wnt5a induces ROR1 dependent NF-κB activation to enhance MMP-9 expression and invasiveness in chronic lymphocytic leukemia

**DOI:** 10.1038/s41375-025-02616-4

**Published:** 2025-04-28

**Authors:** Md Kamrul Hasan, George F. Widhopf II, Emanuela M. Ghia, Thomas J. Kipps

**Affiliations:** Center for Novel Therapeutics, Moores Cancer Center, University of California San Diego, La Jolla, CA USA

**Keywords:** Translational research, Chronic lymphocytic leukaemia

## Abstract

Matrix metalloproteinase-9 (MMP-9) facilitates the extravasation and lymphoid-tissue infiltration of chronic lymphocytic leukemia (CLL) cells. Prior studies found that high level expression of MMP-9 in CLL associates more aggressive disease. We find that circulating CLL cells that express high levels the onco-embryonic protein ROR1 express significantly higher levels of MMP-9. Stimulation of CLL cells with Wnt5a could enhance expression and the release of MMP-9 into the culture media and increase the capacity of CLL cells to invade Matrigel in a Boyden-Chamber Assay. Such effects of Wnt5a could not be inhibited by BTK inhibitors such as ibrutinib or zanubrutinib, but could be blocked by zilovertamab, a humanized mAb specific for ROR1. We found that siRNA silencing of NF-κB-p65 or use of an NF-κB inhibitor (CAS 545380-34-5) blocked the capacity of Wnt5a to induce MMP-9 or enhance the invasive capacity of treated CLL cells. Moreover, siRNA directed silencing of *MMP9* or treatment with an MMP-9 inhibitor (CAS 1177749-58-4) also blocked the invasive capability of CLL cells induced by Wnt5a. We conclude that Wnt5a-induced ROR1-signaling can induce expression of MMP-9 on CLL cells through activation of NF-κB, thereby enhancing the extravasation and lymphoid-tissue infiltration required for CLL cell trafficking.

## Introduction

Chronic lymphocytic leukemia (CLL) is characterized by the accumulation of monoclonal B cells in the marrow and lymphoid tissues, where they receive growth and survival signals within the tumor microenvironment [1, 2]. CLL cells recirculate between blood and lymphoid tissue compartments, and such trafficking requires leukemia cells to pass through endothelial barriers and transverse the extracellular matrix (EM), a process that is facilitated by matrix metallopeptidases (MMP), such as MMP-9 [3]. MMP-9 is a type-IV collagenase and key MMP, which is expressed and released by CLL cells and plays a significant role in pathogenesis [4, 5]. Patients who have CLL cells with high levels of MMP-9 have been found to experience more aggressive disease progression and less favorable clinical outcomes compared to patients who have CLL cells with low-to-negligible MMP-9 [4, 6].

Chemokine-signaling via CXCR4/CXCL12, CXCR5/CXCL13, or CCR7/CCL21 may influence the expression of MMP-9 on CLL cells [7–9]. We examined for this and other factors that might account for the variability in MMP-9 expression among patients with CLL.

One such factor is ROR1. ROR1 is an evolutionarily conserved, type-I membrane protein, expressed on CLL cells, but not on healthy postpartum tissues [10, 11]. It acts as a receptor for Wnt5a, which is elevated in the plasma of CLL patients and can induce non-canonical Wnt signaling [10, 12]. Prior studies found that patients who have CLL cells with high levels of ROR1 had a shorter median time from diagnosis to initial therapy and shorter median overall survival than patients who had CLL cells with low-to-negligible ROR1 [11]. In this study, we compared expression levels of MMP-9 in CLL cells with high versus low-to-negligible ROR1.

We also examined for differences in MMP-9 expression by leukemia cells with or without ROR1 in murine leukemia models. To study the role of ROR1 in leukemogenesis, we previously developed ROR1 transgenic mice and crossed these with Eµ-TCL1 (T-Cell Leukemia 1) TCL1 transgenic (TCL1 Tg) mice to create ROR1xTCL1 double transgenic (ROR1x TCL1 dTg) mice, which demonstrated enhanced leukemogenesis, accelerated disease progression, and reduced survival compared to TCL1 Tg mice [13]. Using the leukemia cells from each of these transgenic mice we examined for MMP-9 in ROR1^+^ leukemia cells of ROR1xTCL1 dTg mice and ROR1^Neg^ leukemia cells from otherwise syngeneic TCL1 Tg mice.

## Materials and methods

### Cell invasion assay

To assess the invasive capacity of CLL cells, we conducted a cell invasion assay. CLL cells (5 × 10^5^) were washed twice and treated with or without Wnt5a (200 ng/ml). The cells were then placed in the top chamber of a Transwell culture polycarbonate insert (Corning, Inc., Corning, NY, USA) with a 6.5-mm diameter and 5 μm pore size, coated with Matrigel. Cells were incubated for 48 h in serum-free medium at 37°C and 5% CO2. The invasion toward the chemokine CXCL12 (200 ng/ml) was analyzed. The percentage of invading cells was calculated as the number of invaded cells in response to the chemokine divided by the total number of input cells.

### ELISA assay

MMP-9 was detected by an MMP-9 ELISA (R & D Systems, Minneapolis, MN, USA). CLL cells (5 × 10^6^/ml) were treated with or without Wnt5a (100 ng/ml) or CXCL12 (100 ng/ml) for 24 h. After centrifugation, supernatants were collected. Diluted supernatants were incubated in MMP-9 Microplates for 2 h at room temperature, followed by the addition of the MMP-9 detector antibody conjugated to HR. The optical density (O.D.) absorbance of each well was measured at 450 nm.

### Co-culture invasion assay

To collect mesenchymal stromal cells (MSCs) conditioned medium (MSC-CM), MSCs isolated from the marrow of CLL patients were cultured at 37°C in a humidified atmosphere containing 5% O_2_ for 3 days. These MSCs constitutively secrete CXCL12 or Wnt5a, as described [14, 15]. Nurse-like cell (NLC) conditioned medium (NLC-CM) was generated using cultures of NLCs isolated from the peripheral blood mononuclear cells (PBMC) of CLL patients, cultured at 37°C and 5% CO_2_, as described [16, 17]. A total of 5 × 10^5^ CLL cells were washed twice, and then were placed into the top chamber of a Transwell culture polycarbonate insert with 6.5-mm diameter and 5 μm of pore size, coated with Matrigel (Corning, Inc., Corning, NY). Cells were incubated for 48 h, and the migration toward MSC-CM or NLC-CM was analyzed. The percentage of migrating cells was calculated as the number of migrated cells in response to MSC-CM or NLC-CM, divided by the total number of input cells.

### CRISPR/CAS9 editing

Single-guide RNAs (sgRNAs) were designed to target Wnt5a using the CHOPCHOP online website. Individual sgRNAs (Invitrogen, Carlsbad, CA) were cloned into the LentiCRISPRv2GFP plasmid (Addgene, Watertown, MA) using the BsmBIv2 restriction enzyme (New England Biolabs, Ipswich, MA). Wnt5a knockout was confirmed via DNA sequencing and Western blot analysis. The sequences of the sgRNAs were AGTATCAATTCCGACATCGA (sense) and TCGATGTCGGAATTGATACT (anti-sense).

### Ethics approval and consent to participate

All methods were performed in accordance with the relevant guidelines and regulations. Blood samples were collected from patients with CLL at the University of California San Diego (UCSD), Moores Cancer Center who satisfied diagnostic criteria for CLL. All patients provided written informed consent, in compliance with the Declaration of Helsinki and the Institutional Review Board (IRB) of the UCSD (IRB approval number 171884). All animal-related experiments were performed in accordance with the National Institutes of Health guidelines for care and use of laboratory animals and in accordance with animal protocols approved by University of California San Diego’s Institutional Animal Care and Use Committee (IACUC) (IACUC approval number S03037).

## Results

### ROR1^Pos^ CLL cells have high-level MMP-9 and enhanced invasive capability compared To CLL cells with low-to-negligible ROR1 (ROR1^Neg^)

**Fig. 1:**
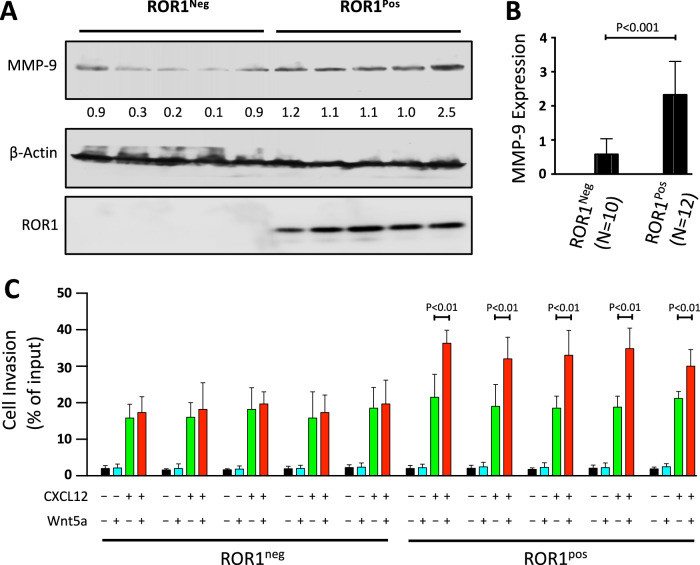
ROR1^Pos^ CLL cells exhibit high-level MMP-9 expression and invasive capability. **A** Immunoblot analysis of lysates prepared from primary ROR1^Pos^ or ROR1^Neg^ CLL cells of different patients; filters were probed with anti-MMP-9, anti-β-actin, or anti-ROR1 antibody as indicated on the left. The numbers between two lanes are ratios of band integrated optical density (IOD) of MMP-9 versus total β-actin. **B** Expression of MMP-9 was assessed by immunoblot analysis of lysates prepared from primary CLL cells of different patients with CLL cells that did (ROR1^Pos^ [*N* = 12]) or did not (ROR1^Neg^ [*N* = 10]) express ROR1. The ratios of band IOD of MMP-9 versus total β-actin were determined and plotted in the graph. Data are shown as mean ± SD. *P* < 0.001, as assessed by two-tailed Student’s *t-*test. **C** ROR1^Pos^ or ROR1^Neg^ CLL-cell invasion in response to CXCL12 (200 ng/ml) was assessed without (−) or with (+) exogenous Wnt5a (200 ng/ml), as indicated at the bottom. Data are shown as mean ± SD from three independent experiments. *p* < 0.01, as assessed by two-tailed Student’s *t-*test.

Previous studies demonstrated that MMP-9 can be expressed by primary CLL cells and that this contributes to pathogenesis [5]. Additionally, patients who have CLL cell with high MMP-9 have been found to have more aggressive disease progression and less favorable clinical outcomes compared to patients who have CLL cells with low-to-negligible MMP-9 [4]. Using immunoblot analyses we examined the levels of MMP-9 in CLL cells with low-to-negligible ROR1 (ROR1^Neg^) and in CLL cells with high levels of ROR1 (ROR1^Pos^), as defined in a prior study (Supplementary Table 1) [11]. We found that ROR1^Pos^ CLL cells of different patients (*N* = 12) had significantly higher levels of MMP-9 than ROR1^Neg^ CLL cells from each of 10 different patients (*p* < 0.001) (Fig. 1A, B).

We then analyzed the functional role of MMP-9 in degrading Matrigel-coated membranes and in influencing the invasive capability of CLL cells. Consistent with previous studies [7], CLL cells invaded Matrigel-coated membranes in response to the chemokine CXCL12, a ligand of CXCR4 expressed on CLL cells (Supplementary Fig. S1). ROR1^Pos^ CLL cells of different patients (*N* = 5) showed enhanced invasive capability to CXCL12 when stimulated with Wnt5a (Supplementary Fig. S1). In a cell invasion assay using ROR1^Pos^ and ROR1^Neg^ CLL cells, both types exhibited invasive capability in response to CXCL12. However, Wnt5a stimulation enhanced the invasive capacity of ROR1^Pos^ CLL cells but not ROR1^Neg^ CLL cells (Fig. 1C).

We examined the leukemia cells derived from TCL1 Tg or ROR1xTCL1 dTg mice for mouse MMP-9. The leukemia cells of TCL1-Tg mice lack ROR1, whereas the leukemia cells of ROR1xTCL1 dTg mice express human ROR1 at levels comparable to that of ROR1^Pos^ CLL [13]. We found that splenic leukemia cells of ROR1xTCL dTg mice have higher levels of MMP-9 than the similarly isolated splenic leukemia cells of TCL1 Tg mice (Supplementary Fig. S2A). We also performed a cell invasion assay using splenic leukemia cells of TCL1 Tg or ROR1xTCL1 dTg mice in response to mouse CXCL12, without or with stimulation of Wnt5a. Our findings indicate that both TCL1 and ROR1xTCL1 leukemia cells possess invasive capabilities in response to CXCL12. However, stimulation with Wnt5a significantly increased the invasive capacity of ROR1xTCL1 splenic leukemia cells compared to TCL1 leukemia cells (Supplementary Fig. S2B).

### Wnt5a enhances MMP-9 expression and invasive capability via ROR1 dependent mechanism

**Fig. 2:**
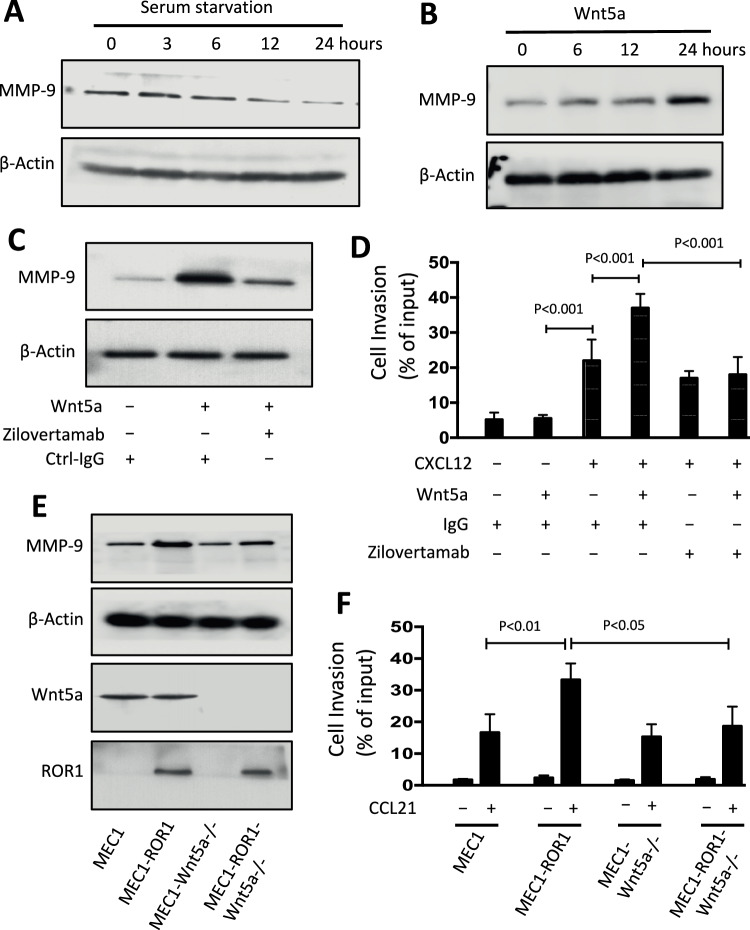
Wnt5a contributes MMP-9 expression and invasive capacity via ROR1 dependent mechanism. **A** Immunoblot analysis of lysates prepared from freshly-isolated primary CLL cells that were serum-starved for the times indicated on the top (in hours); the filters were probed with MMP-9, or β-actin antibody, as indicated on the left. **B** Immunoblot analysis of lysates prepared from overnight, serum-starved primary CLL cells that subsequently were treated without (−) or with (+) Wnt5a (100 ng/ml), for the times indicated on the top (in hours); the filters were probed with MMP-9 or β-actin antibody, as indicated on the left. **C** Immunoblot analysis of lysates prepared from overnight, serum-starved primary CLL cells that subsequently were treated with Ctrl-IgG or zilovertamab (20 μg/ml), without (−) or with (+) Wnt5a (100 ng/ml), as indicated at the bottom; the filters were probed with MMP-9 or β-actin antibody, as indicated on the left. **D** To CLL cells we added Ctrl-IgG or zilovertamab (20 μg/ml) without (−) or with (+) Wnt5a (200 ng/ml), and examined the treated cells for their ability to invade Matrigel in response to CXCL12 (200 ng/ml). Each bar depicts the mean ± SD of each culture condition as indicated at the bottom of the histogram from three independent experiments of CLL cells from each of six patients. *p* < 0.001, as determined by two-tailed Student’s *t*-test. **E** Lysates were prepared from MEC1, MEC1-ROR1, or MEC1-Wnt5a ^−/−^, and MEC1-ROR1-Wnt5a ^−/−^ cells for immunoblot analysis; the filters were probed with antibodies specific for MMP-9, ROR1, Wnt5a, or β-actin, as indicated on the left of the immunoblot. **F** MEC1, MEC1-ROR1, MEC1 -Wnt5a ^−/−^, or MEC1-ROR1-Wnt5a ^−/−^ cells were examined for their relative capacity to invade Matrigel without  (−) or with (+) CCL21 (200 ng/ml) as indicated at the bottom of the histogram. Each bar depicts the mean ± SD from three independent experiments (*n* = 3). *p* < 0.05; *p* < 0.01, as calculated using two-tailed Student’s *t-*test.

We examined the levels of MMP-9 in CLL cells after culture with or without of Wnt5a. ROR1^Pos^ CLL cells (*N* = 3) cultured without Wnt5a exhibited a decrease in MMP-9 levels over time (Fig. 2A). Conversely, the addition of exogenous Wnt5a led to a time-dependent increase in MMP-9 (Fig. 2B). However, pre-treatment with zilovertamab, an anti-ROR1 antibody, inhibited the capacity of Wnt5a to induce ROR1^Pos^ CLL cells to increase MMP-9, suggesting that this effect was mediated via ROR1-signaling (Fig. 2C). In a cell invasion assay, Wnt5a augmented CLL cell invasiveness in response to CXCL12, but this enhancement was blocked by pre-treatment with zilovertamab (Fig. 2D).

We examined whether Wnt5a also could enhance CXCR5/CXCL13-induced invasiveness of CLL cells. ROR1^Pos^ CLL cells (*N* = 6) cultured with CXCL13 had increased invasiveness, which was further enhanced by addition of Wnt5a. Zilovertamab pre-treatment inhibited the capacity of Wnt5a to enhance invasiveness, suggesting that this capacity was dependent on ROR1-signaling (Supplementary Fig. S3).

We corroborated these findings using a CLL-derived cell line, MEC1 [18]. We previously identified that MEC1 cells expressed Wnt5a, but not ROR1 [19]. We transduced MEC1 cells with a lentivirus vector driving expression of human ROR1 to generate MEC1 cells that expressed high levels of surface ROR1 (designated MEC1-ROR1). We found that MEC1-ROR1 cells expressed significantly higher levels of MMP-9 than MEC1 cells (Fig. 2E). In addition, we disrupted both alleles of Wnt5a via CRISPR-Cas9 in MEC1 to generate MEC1-Wnt5a^−/−^ cells, which lacked expression of Wnt5a. We also transduced these cells with the ROR1-expression vector to generate MEC1-ROR1-Wnt5a^−/−^ cells, which also lacked expression of Wnt5a. We found that MEC1-ROR1-Wnt5a^−/−^ cells expressed levels of MMP-9 comparable to that of MEC1 cells. Moreover, the levels of MMP-9 expressed by either of these cell lines were significantly lower than that of MEC1-ROR1 cells. (Fig. 2E). We also performed cell invasion assays to examine the capacity of MEC1, MEC1-ROR1, MEC1-Wnt5a^−/−^, or MEC1-ROR1-Wnt5a^−/−^ cells to migrate and invade Matrigel in response to CCL21, a chemokine for CCR7, which is expressed by MEC1 cells. This study demonstrated that MEC1-ROR1 cells had significantly higher invasiveness than either MEC1 cells or MEC1-ROR1-Wnt5a^−/−^ cells (Fig. 2F). These results indicate that Wnt5a and ROR1 are required for the enhanced invasiveness of MEC1-ROR1 cells relative to MEC1 cells.

High MMP-9 levels are observed in the serum of CLL patients [4, 6]. We investigated whether Wnt5a/ROR1 signaling in CLL cells enhanced the release of MMP-9 into the culture media, as assessed via an ELISA for MMP-9. We cultured ROR1^Pos^ CLL cells (*N* = 5) in media with or without Wnt5a. We found that treatment with Wnt5a enhanced the release of MMP-9 into the culture medium (Supplementary Fig. S4A). On the other hand, pre-treatment of the CLL cells with zilovertamab prior to culture blocked the capacity of Wnt5a to enhance release of MMP-9 into the media. In contrast, pre-treatment of the CLL cells with ibrutinib, a BTK inhibitor that blocks CXCR4 signaling, did not inhibit the capacity of Wnt5a to enhance release of MMP-9, indicating that Wnt5a-induced ROR1-signaling promotes expression and release of MMP-9 independent of BTK or CXCR4 signaling. To confirm that the ibrutinib treatment was effective, we similarly treated CLL cells with or without ibrutinib prior to culture in media with or without the chemokine CXCL12, which can enhance expression and release of MMP-9 from CLL cells [7, 20, 21]. We noted that ibrutinib could block the capacity of CXCL12 to enhance release of MMP-9 into the culture media (Supplementary Fig. S4B).

### MMP-9 Is necessary for Wnt5a/ROR1 enhanced invasiveness of CLL cells

**Fig. 3:**
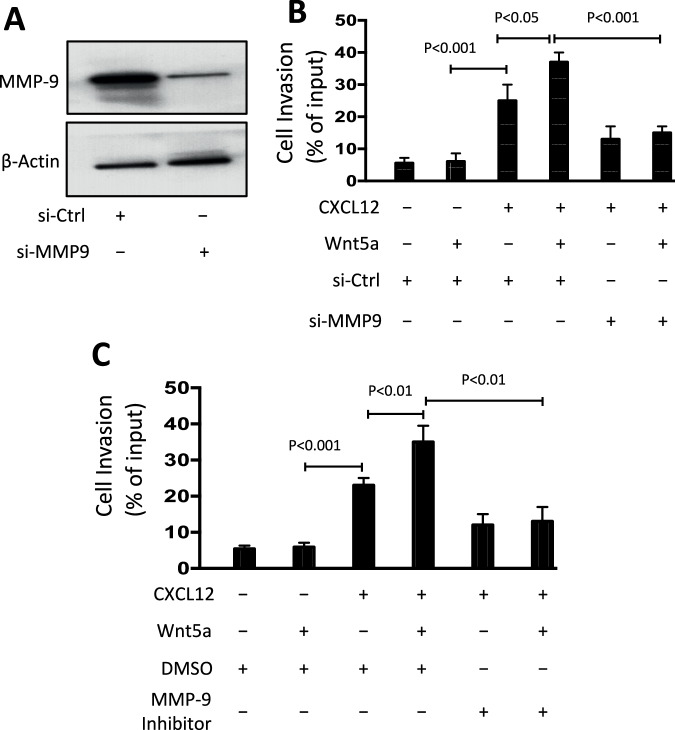
MMP-9 is necessary for Wnt5a/ROR1 enhanced invasive capability of CLL cells. **A** Immunoblot analysis of lysates prepared from CLL cells transfected 72 h before with control siRNA or siRNA targeting MMP-9; filters were probed with antibodies specific for MMP-9 or β-actin, as indicated on the left. Cell viability was over 85% in control-siRNA and MMP-9-siRNA transfected cells. **B** CLL-cell invasion in response to CXCL12 (200 ng/ml) was assessed without (−) or with (+) exogenous Wnt5a (200 ng/ml), as indicated at the bottom of the histogram. Each bar represents the mean percent cell invasion (± SD) of each culture condition in each of three independent experiments of CLL cells from each of six patients. *p* < 0.05; *p* < 0.001, as assessed by two-tailed Student’s *t-*test. **C** CLL-cell invasion in response to CXCL12 (200 ng/ml) was assessed by adding an MMP-9 inhibitor (CAS 1177749-58-4; 10 nM) to the cells prior to treatment without (−) or with (+) Wnt5a (200 ng/ml), as indicated at the bottom of the histogram. Each bar depicts the mean percent cell invasion ( ± SD) in each culture condition in three independent experiments of CLL cells from each of six patients. *p* < 0.01; *p* < 0.001, as assessed by two-tailed Student’s *t-*test.

We conducted an immunoblot analyses of cell lysates isolated from CLL cells transfected with either control siRNA or siRNA targeting *MMP9*. The results showed reduced MMP-9 expression in cells transfected with *MMP9* siRNA compared to CLL cells transfected with non-specific control siRNA (Fig. 3A). In a subsequent cell invasion assays, CLL cells were exposed to CXCL12 with or without Wnt5a. We found that treatment with siRNA targeting *MMP9*, but not the control siRNA, suppressed the ability of Wnt5a to enhance chemokine-directed CLL cell invasion (Fig. 3B). Furthermore, pre-treatment with an MMP-9 specific inhibitor (CAS 1177749-58-4) blocked the capacity of Wnt5a to enhance the invasiveness of CLL cells, indicating that MMP-9 plays a crucial role in Wnt5a/ROR1-enhanced CLL cell invasion (Fig. 3C).

### Zilovertamab, but not BTK inhibitors, could block the capacity of Wnt5a To enhance CLL MMP-9 expression and invasiveness

**Fig. 4:**
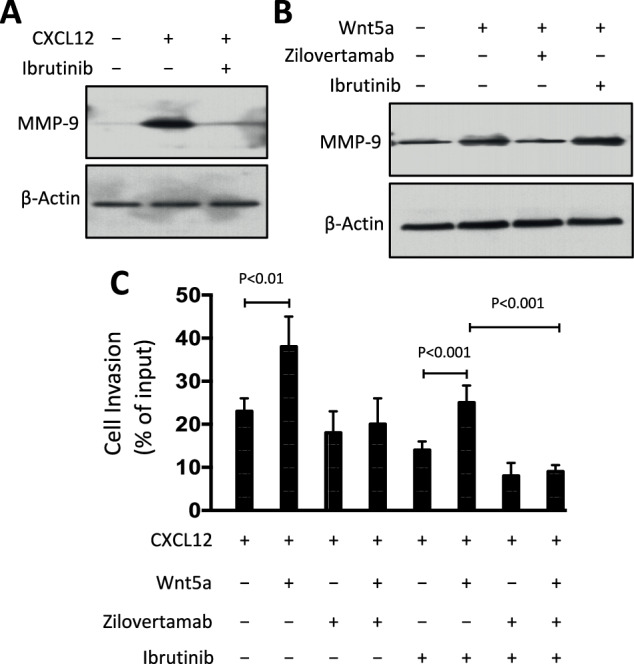
Zilovertamab but not ibrutinib could block Wnt5a enhanced MMP-9 expression and invasive capability of CLL cells. **A** Lysates for immunoblot analyses were prepared from serum-starved primary CLL cells that were treated without (−) or with (+) ibrutinib (0.5 μm), without (−) or with (+) CXCL12 (100 ng/ml), as indicated on the top of the immunoblot; the filters were probed with antibodies specific for MMP-9 or β-actin, as indicated on the left. **B** Lysates were prepared from serum-starved primary CLL cells that were treated with Ctrl-IgG or zilovertamab (20 μg/ml), without (−) or with (+) Wnt5a (100 ng/ml) or ibrutinib (0.5 μm), as indicated at the top of the immunoblot; the filters were probed with antibodies to MMP-9 or β-actin, as indicated on the left. **C** To CLL cells we added Ctrl-IgG or zilovertamab (20 μg/ml) or ibrutinib (0.5 μm) prior to treatment without (−) or with (+) Wnt5a (200 ng/ml) and examined the treated cells for their capacity to invade Matrigel in response to CXCL12 (200 ng/ml), as indicated at the bottom of the histrogram. Each bar depicts the  mean percent invasion (± SD) of cells treated in each culture condition as indicated at the bottom of the histogram. The data are from three independent experiments of CLL cells from each of six patients. *p* < 0.01; *p* < 0.001, as determined by two-tailed Student’s *t-test*.

We performed immunoblot analyses on cell lysates of CLL cells stimulated with or without CXCL12 and noted that CXCL12 also enhanced expression of MMP-9 (Fig. 4A), as noted in prior studies [7]. As expected, pre-treatment with ibrutinib, or another BTK inhibitor, zanubrutinib [22], blocked the capacity of CXCL12 to enhance expression of MMP-9 (Fig. 4A) [20]. Such treatment, however, could not inhibit the capacity of Wnt5a to enhance expression of MMP-9 (Fig. 4B and Supplementary Fig. S5). On the other hand, pre-treatment with the anti-ROR1 mAb zilovertamab could inhibit the capacity of Wnt5a to enhance MMP-9 (Fig. 4B).

We also assessed the invasive capacity of CLL cells treated with CXCL12 in media with or without Wnt5a and with or without pre-treatment with zilovertamab, ibrutinib, or zanubrutinib. Zilovertamab blocked Wnt5a-enhanced invasiveness, but ibrutinib or zanubrutinib did not (Fig. 4C and Supplementary Fig. S6). This suggests a potential mechanism that could contribute to resistance to BTK inhibitor therapy of patients with CLL. Combined therapy with zilovertamab and ibrutinib showed additive efficacy in inhibiting CLL cell invasiveness, indicating that these agents have complementary inhibitory activity.

### Wnt5a/ROR1 signaling induces NF-κB activation to enhance expression of MMP-9 In CLL

**Fig. 5:**
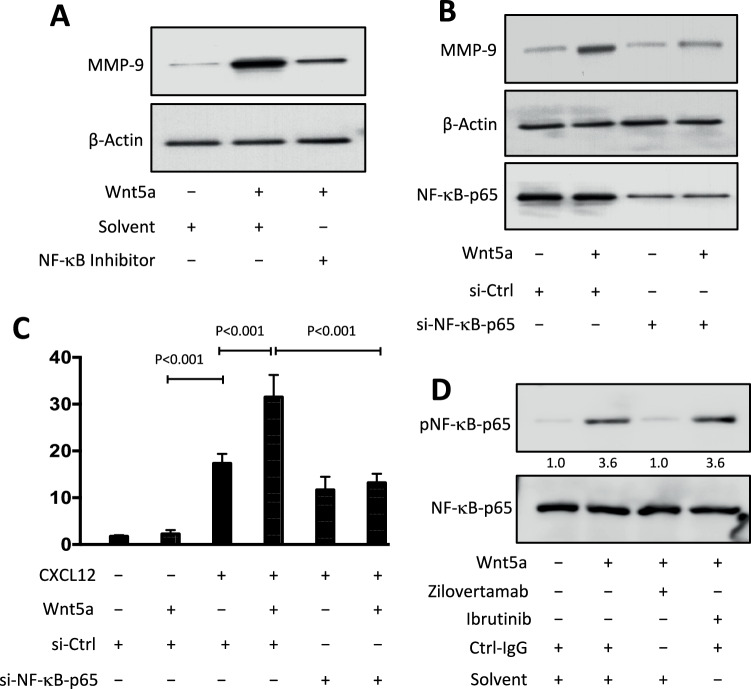
Wnt5a/ROR1 signaling induces NF-κB activation to enhance MMP-9 expression in CLL. **A** Lysates were prepared from serum-starved primary CLL cells that subsequently were treated without (–) or with (+) NF-κB inhibitor (CAS 545380-34-5, 20 nM), prior to treatment without (–) or with (+) Wnt5a (100 ng/ml), as indicated at the bottom of the immunoblot; the filters were probed with antibodies specific for MMP-9 or β-actin, as indicated on the left. **B** CLL-cells were treated without (–) or with (+) Wnt5a (100 ng/ml) 72 hours after they were transfected with control siRNA or siRNA targeting NF-κB-p65. Lysates were prepared for immunoblot analyses from each culture condition as indicated at the bottom of the immunoblots, which were probed with antibodies specific for MMP-9, NF-κB-p65, or β-actin, as indicated on the left. **C** 72 hours after transfection with control siRNA or siRNA targeting NF-κB-p65, CLL cells were examined for their ability to invade Matrigel without (–) or with (+) Wnt5a in response to CXCL12 (200 ng/ml). Cell viability was over 85% in control- and NF-κB-p65-siRNA transfected CLL cells. Each bar depicts the mean percent cell invasion (± SD) of the CLL cells in each culture condition indicated at the bottom from three independent experiments of CLL cells from each of six patients. *p* < 0.001, as assessed by two-tailed Student’s *t-*test. **D** Immunoblot analysis of lysates prepared from serum-starved primary CLL cells that were treated with Ctrl-IgG or zilovertamab (20 μg/ml), or ibrutinib (0.5 μm), prior to treatment without (–) or with (+) Wnt5a (100 ng/ml), as indicated at the bottom; the filters were probed with antibodies to NF-κB-p65 or pNF-κB-p65, as indicated on the left.

The gene promoter for *MMP9* contains an NF-κB binding site [23, 24]. We investigated whether activation of NF-κB induced by ROR1-signaling accounts for its capacity to enhance expression of MMP-9. We performed immunoblot analyses for MMP-9 using lysates prepared from CLL cells stimulated without or with Wnt5a, and without or with an inhibitor of NF-κB (CAS 545380-34-5) (Fig. 5A). We found that CAS 545380-34-5 could inhibit the capacity of Wnt5a to enhance CLL cell expression of MMP-9.

We also transfected CLL cells with siRNA targeting NF-κB-p65, which reduced CLL cell expression of NF-κB-p65, compared to that of CLL cells transfected with a non-specific siRNA (Fig. 5B). CLL cells silenced for NF-κB-p65 also were not induced by Wnt5a to express higher levels of MMP-9, in contrast to the CLL cells treated with the control siRNA. CLL cells silenced for NF-κB-p65 also did not have enhanced invasiveness in response to Wnt5a (Fig. 5C). This was not the case for CLL cells treated with the control siRNA. Furthermore, when CLL cells were pre-treated with zilovertamab, Wnt5a was not able to induce phosphorylation of NF-κB-p65 (Fig. 5D). On the other hand, treatment with ibrutinib did not inhibit the capacity of Wnt5a to induce CLL-cell phosphorylation of NF-κB-p65 (Fig. 5D).

### Coculture of CLL cells with marrow mesenchymal stromal cells (MSCs) can enhance MMP-9 expression and invasiveness of CLL cells

**Fig. 6:**
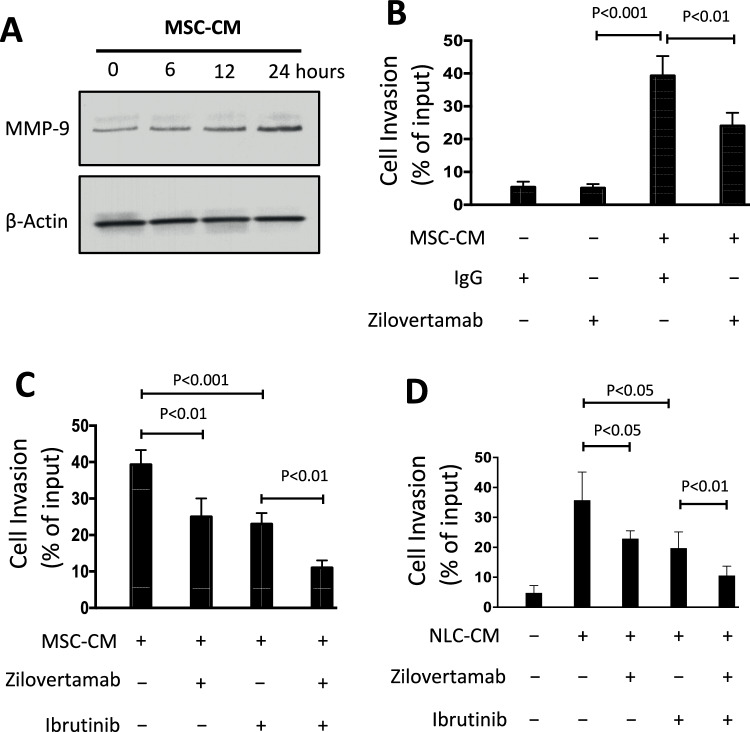
Culture of CLL cells in MSC-conditioned or NLC-conditioned media enhanced their expression of MMP-9 and invasiveness. **A** Immunoblot analysis of lysates prepared from serum-starved primary CLL cells that were treated without (–) or with (+) MSC-CM, for the times indicated on the top (in hours); the filters were probed with antibodies specific for MMP-9 or β-actin, as indicated on the left. **B** We treated CLL cells with zilovertamab (20 μg/ml) or Ctrl-IgG, and cultured the cells for 48 hours in MSC-CM or non-conditioned media. The cells in each culture condition (as indicated at the bottom of the histogram) were examined for their capacity to invade Matrigel. The bars depict the mean percent cell invasion (± SD) from 3 independent experiments using CLL cells from each of six patients. *p* < 0.01; *p* < 0.001, as determined by two-tailed Student’s *t-*test. **C** We treated CLL cells with zilovertamab (20 μg/ml), Ctrl-Ig, or ibrutinib (0.5 μm), and cultured the cells for 48 hours in MSC-CM. We then determined their percent Matrigel invasion capacity, as indicated on the left. Each bar depicts the mean percent invasion capacity (±SD) of cells treated as indicated at the bottom in three independent experiments of CLL cells from each of six patients. *p* < 0.01; *p* < 0.001, as determined by two-tailed Student’s *t-*test. **D** We treated CLL cells with Ctrl-IgG or zilovertamab (20 μg/ml), or ibrutinib (0.5 µm), prior to culture in NLC-CM and then examined their capacity to invade Matrigel, as indicated at the bottom. Each bar depicts the mean percent invasion capacity (±SD) of CLL cells treated as indicated at the bottom from three independent experiments of CLL cells from each of six patients. *p* < 0.05; *p* < 0.01, as determined by two-tailed Student’s *t-*test.

We described previous studies involving the in-vitro culture of marrow mesenchymal stromal cells (MSCs) at physiologic oxygen tension (e.g., 5% O_2_ in N_2_) [14]. Under such conditions, MSC cells can release growth or survival factors, such as CXCL12 or Wnt5a, into the culture media [14, 15]. We found that culture of CLL cells in MSC-conditioned media (MSC-CM) enhanced leukemia-cell expression of MMP-9 in a time dependent manner (Fig. 6A). We also found that CLL cells cultured in MSC-CM had greater capacity to invade Matrigel than CLL cells cultured in non-conditioned media. However, we found that CLL cells treated with zilovertamab, but not Ctrl-IgG, for 2 hours prior to culture inhibited the capacity of MSC-CM to enhance the invasiveness of CLL cells (Fig. 6B). Treatment of CLL cells with ibrutinib also blocked the capacity of MSC-CM to enhance the invasiveness of CLL cells (Fig. 6C). Moreover, combined treatment with zilovertamab and ibrutinib had even greater capacity to inhibit the capacity of MSC-CM to enhance CLL cell invasiveness (Fig. 6C).

We obtained similar results using nurse like cells (NLCs), which also release Wnt5a and CXCL12 into the cultured media [17, 25]. We found that co-culture with NLCs could enhance the invasiveness of CLL cells treated with non-specific antibody, but not of CLL cells pretreated with zilovertamab (Fig. 6D). Moreover, combined therapy of zilovertamab and ibrutinib had additive activity in inhibiting the enhanced invasiveness of CLL cells co-cultured with NLCs (Fig. 6D).

## Discussion

Our study uncovered a novel mechanism underlying CLL cell upregulation of MMP-9, which prior studies found at higher levels in the plasma of CLL patients compared to healthy individuals [21]. We observed that serum starvation of CLL cells led to a downregulation of MMP-9 expression, whereas stimulation with Wnt5a enhanced expression of MMP-9. Additionally, Wnt5a increased the chemokine-directed invasiveness of CLL cells. Pre-treatment with zilovertamab, an anti-ROR1 antibody, blocked Wnt5a’s ability to increase MMP-9 expression and enhance the invasiveness of CLL cells. Zilovertamab also inhibited the enhanced invasiveness of CLL cells cultured in MSC- or NLC-conditioned medias. Furthermore, lowering expression of MMP-9 or inhibiting its function using an MMP-9 inhibitor reduced the invasiveness of CLL cells, indicating the capacity of Wnt5a/ROR1-signaling to enhance invasiveness of CLL cells is dependent on MMP-9.

In leukemia B cells, we found that Wnt5a could upregulate MMP-9 expression via ROR1-dependent activation of NF-κB. It should be noted that other signaling molecules, such as CXCL12 (a ligand for CXCR4), can enhance MMP-9 expression and migration, as well as the invasive capacity of CLL cells [7]. CXCL12/CXCR4 signaling upregulates MMP-9 via the ERK1/2/c-Fos signaling pathways. Similarly, CXCL13/CXCR5 signaling can upregulate MMP-9 through the RANKL-Src axis, which other studies showed could facilitate lymph node metastasis in breast cancer [9]. Another chemokine, CCL21, a ligand for CCR7, also upregulates MMP-9 via ERK1/2/c-Fos pathways [8]. In contrast, integrin-mediated CLL cell adhesion can upregulate MMP-9 through the PI3K/Akt/NF-κB pathway [7]. Shin and colleagues also reported that phorbol myristate acetate (PMA) could induce MMP-9 expression via a protein kinase Cα (PKCα)-NF-κB dependent signaling cascade in BEAS-2B human lung epithelial cells [26]. Moreover, 2-chloroethanol (2-CE) treatment upregulates MMP-9 via p38 mitogen-activated protein kinase (p38 MAPK) signaling pathway through activation of both NF-κB and AP-1 in rat astrocytes. Our findings indicate that MMP-9 expression can be influenced by signaling pathways that operate independently of each other in the same neoplastic cell population. Therefore, inhibiting just one pathway may not be sufficient to fully suppress expression of MMP-9.

The promoter region of *MMP9* includes an NF-κB binding site [23]. Research by Rhee and colleagues demonstrated that NF-κB activation is crucial for lipopolysaccharide (LPS)-induced MMP-9 expression in the RAW 264.7 macrophage cell line [27]. They cloned a segment of the *MMP9* promoter spanning from -664 to +75 base pairs, which exhibited increased promoter activity following LPS stimulation. A chromatin immunoprecipitation (ChIP) assay confirmed that NF-κB-p65 binds to the *MMP9* promoter. However, when the NF-κB binding site was inactivated via gene disruption, LPS failed to enhance *MMP9* promoter activity, indicating that NF-κB-p65 binding is necessary for *MMP9* expression [27]. Our findings indicate that Wnt5a can activate NF-κB, which may then bind to the *MMP9* promoter and upregulate expression of MMP-9 in CLL cells [17].

The ability of CLL cells to traffic within the body is a crucial factor in disease progression. A hallmark of CLL is the homing and infiltration of leukemia cells into lymphoid tissues, including marrow, lymph nodes, and spleen, which provides a favorable environment for CLL cell growth and survival. Notably, CLL cells within these tissues express higher levels of MMP-9 compared to those in peripheral blood [8]. While CLL cells can release MMP-9, it has also been detected on their plasma membrane [4, 28]. Specifically, MMP-9 can bind to a docking complex formed by α4β1 integrin and 190 kDa CD44v on the CLL cell surface, potentially enhancing its catalytic activity and facilitating the invasiveness of CLL cells [28]. Upon adhesion to substrates like fibronectin, VCAM-1, or FN-H89, CLL cells form podosomes, where MMP-9 localizes and contributes to the degradation of extracellular matrices through a PI3K activation pathway. Our study reveals that the Wnt5a/ROR1 signaling pathway upregulates expression and release of MMP-9, thereby enhancing the invasiveness of CLL cells. This mechanism suggests a role for ROR1 in accelerating CLL disease progression [11].

We have previously explored the roles of various cytoplasmic proteins, including HS1, cortactin, DOCK2, and 14-3-3ζ, in enhancing the migration and proliferation of CLL cells [19, 29–31]. For instance, HS1 and cortactin bind to ROR1 at proline-841, recruit ARHGEF1, to activate RhoA, leading to increased F-actin polymerization and migration in CLL cells [29, 31, 32]. Similarly, DOCK2 interacts with ROR1 at proline-808, activating Rac1 and ERK1/2 to promote CLL cell proliferation [30, 33]. Additionally, 14-3-3ζ associates with ROR1 at serine-867, recruits ARHGEF2, and activates both RhoA and Rac1, further enhancing CLL cell migration and proliferation [19]. Despite this understanding, the impact of Wnt5a/ROR1 signaling on degrading the Matrigel membrane remained unexplored. Our study addresses this gap by providing the first evidence that Wnt5a/ROR1 signaling upregulates MMP-9, facilitating the degradation of the Matrigel membrane and contributing to the invasiveness of CLL cells—a factor associated with poor clinical outcomes in CLL patients [4, 6].

We propose that Wnt5a significantly contributes to the upregulation of MMP-9 through NF-κB activation, thereby enhancing the invasiveness and disease progression of CLL. This effect of Wnt5a is dependent on ROR1 and can be inhibited by zilovertamab, a first-in-class anti-ROR1 antibody that has been found safe for treating patients with CLL [34]. Our findings indicate that BTK inhibitors, such as ibrutinib or zanubrutinib, fail to inhibit Wnt5a-induced NF-κB activation or expression of MMP-9, suggesting a potential mechanism for drug resistance and disease progression in CLL. Notably, combining therapies showed enhanced efficacy in blocking the invasiveness of CLL cells, highlighting a potential benefit of combined therapy of zilovertamab with BTK inhibitors for patients with CLL.

## Supplementary information

Supplementary Information

Supplementary Figures

## Data Availability

Data files will be made available upon reasonable request to the corresponding authors.
