# Supplementary material for: Wnt5a induces ROR1 dependent NF-κB activation to enhance MMP-9 expression and invasiveness in chronic lymphocytic leukemia
Source: Leukemia. 2025 Apr 28;39(7):1661–9. doi: 10.1038/s41375-025-02616-4 (PMC12208867; doi:10.1038/s41375-025-02616-4)
Supplement: Supplementary file 1 — Supplementary Information [file 41375_2025_2616_MOESM1_ESM.docx]

**Supplementary Information**

**Wnt5a Induces ROR1 Dependent NF-κB Activation To Enhance MMP-9 Expression And Invasiveness In Chronic Lymphocytic Leukemia**

Md Kamrul Hasan^1^, George F. Widhopf II^1^, Emanuela M. Ghia^1^, and Thomas J. Kipps^1^

^1^Center for Novel Therapeutics, Moores Cancer Center, University of California San Diego, La Jolla, California, USA.

**Supplementary Materials and Methods**

**Cell culture and CLL specimens**

MEC1 cells were cultured in RPMI medium with 10% FBS, 1% Penicillin/Streptomycin and maintained at 37 ̊C in a humidified atmosphere of 5% CO2, and tested negative for Mycoplasma contamination. Media and supplements were purchased from Life Technologies (Carlsbad, CA, USA). Blood samples were collected from CLL patients at the Moores Cancer Center. Peripheral blood mononuclear cells (PBMCs) were isolated by density centrifugation with Ficoll-Paque PLUS (GE Healthcare Life Sciences) and suspended in 90% FBS (Omega Scientific) and 10% DMSO (Sigma-Aldrich) for viable storage in liquid nitrogen.

**Immunoblot analysis**

Equal amounts of total protein from each sample were separated by sodium dodecyl sulfate-polyacrylamide gel electrophoresis and blotted onto polyvinylidene difluoride membrane. Western blot analysis was performed using primary mAbs specific for MMP-9 (Cell Signaling, Cat#13667; or R&D Systems, Cat#AF909), NF-κB-p65 (Cell Signaling, Cat#8242), phospho pNF-κB-p65 (Cell Signaling, Cat#3033), ROR1 (Cell Signaling, Cat# 4102), Wnt5a (R&D Systems, Minneapolis, MN, Cat#MAB645) or β-Actin (Cell Signaling, Cat# 4967), which were detected using secondary antibodies conjugated with horse- radish peroxidase (Cell Signaling Technology, Danvers, MA).

**Nucleofection of plasmids and siRNAs**

The Human B Cell Nucleofector Kit for siRNA or plasmid transfection (Lonza, Basel, Switzerland) was used. B-CLL cells or MEC1 cells (5 × 10^6^) were suspended in 100 μl Nucleofector Solution with plasmids or siRNAs (Life Technologies, Carlsbad, CA) and transfected with the Nucleofector II device (program U-015). The transfected cells were cultured in 12-well plates in complete medium for 48 hours (plasmids) or 72 hours (siRNAs) and then subjected to immunoblot analysis and assays.

**Mice Study**

We previously generated C57BL/6 transgenic (Tg) mice with human ROR1 regulated by the murine Ig promoter/enhancer, leading to B-cell–restricted expression of ROR1.^1^ ROR1 × TCL1 double transgenic (dTg) mice were generated by mating ROR1 Tg mice with Eµ-TCL1 Tg mice.^2^

In contrast to leukemia cells that develop in TCL1-Tg mice, the leukemia cells that develop in double-Tg ROR1×TCL1 dTg mice express human ROR1. ROR1×TCL1 leukemia B cells (CD5+B220loROR1+) and TCL1 leukemia cells (CD5+B220loROR1-) were isolated from the spleen, enriched via density gradient centrifugation, suspended in sterile PBS and verified by multiparametric flow cytometry to be more than 90% leukemia B cells.

### **Statistical analysis**

### Data are presented as mean ± SD. Differences between two groups were determined by unpaired two-tailed Student’s t-test. All P values of less than 0.05 were considered significant. Analysis for significance was performed with GraphPad Prism 6.0 (GraphPad Software Inc.).

**Supplementary Figure Legends**

**Supplementary Fig. S1.** ROR1^Pos^ CLL-cell invasion in response to CXCL12 (200 ng/ml) was assessed without (−) or with (+) exogenous Wnt5a (200 ng/ml), as indicated at the bottom. Data are shown as mean ± SD from three independent experiments. *p* < 0.001, as assessed by two-tailed Student’s *t-*test.

**Supplementary Fig. S2.** (**A**) Immunoblot analysis of lysates prepared from splenic leukemia cells that were isolated using Ficoll-Paque PLUS density gradient media (Cytiva) of TCL1 Tg mice or ROR1xTCL1 dTg mice, as indicated on the top; the filters were probed with MMP-9, ROR1 or β-actin antibody, as indicated on the left. (**B**) TCL1 or ROR1xTCL1 splenic leukemia cell invasion in response to mouse CXCL12 (200 ng/ml) was assessed without (−) or with (+) exogenous Wnt5a (200 ng/ml), as indicated at the bottom. Data are shown as mean ± SD from three independent experiments. *p* < 0.01, as assessed by two-tailed Student’s *t-*test.

**Supplementary Fig. S3.** CLL cells were added Ctrl-IgG or zilovertamab (20 μg/ml) prior to treatment without (−) or with (+) Wnt5a (200 ng/ml), and examine for cell invasion in response to CXCL13 (200 ng/ml), as indicated at the bottom. Data are shown as mean ± SD from three independent experiments of CLL cells from each of six patients. *p* < 0.01; *p* < 0.001, as determined by two-tailed Student’s *t*-test.

**Supplementary Fig. S4.** (**A**) CLL cells were serum-starved overnight and added Ctrl-IgG or zilovertamab (20 μg/ml) or ibrutinib (0.5 μm) prior to stimulated without (−) or with (+) Wnt5a (100 ng/ml), and examined release for MMP-9 (ng/ml) to the cultured media by ELISA assay, as indicated at the bottom. Data are shown as mean ± SD from three independent experiments of CLL cells from each of five patients. *p* < 0.01; *p* < 0.001, as determined by two-tailed Student’s *t-*test. (**B**) CLL cells were serum-starved overnight and treated without (−) or with (+) ibrutinib (0.5 μm) prior to stimulated without (−) or with (+) CXCL12 (100 ng/ml), and examine release for MMP-9 (ng/ml) to the cultured media by ELISA assay, as indicated at the bottom. Data are shown as mean ± SD from three independent experiments of CLL cells from each of five patients. *p* < 0.01; *p* < 0.001, as determined by two-tailed Student’s *t-*test.

**Supplementary Fig. S5.** Immunoblot analysis of lysates prepared from overnight, serum-starved primary CLL cells that subsequently were treated with Ctrl-IgG or zilovertamab (20 μg/ml), without (−) or with (+) zanubrutinib (0.5 μm), without (−) or with (+) Wnt5a (100 ng/ml), as indicated at the bottom; the filters were probed with MMP-9 or β-actin antibody, as indicated on the left.

**Supplementary Fig. S6.** CLL cells were added Ctrl-IgG or zilovertamab (20 μg/ml) or zanubrutinib (0.5 μm) prior to treatment without (−) or with (+) Wnt5a (200 ng/ml) and examined for cell invasion in response to CXCL12 (200 ng/ml), as indicated at the bottom. Data are shown as mean ± SD from three independent experiments of CLL cells from each of six patients. *p* < 0.01, as determined by two-tailed Student’s *t-test*.

**References**

1 Widhopf, G. F., 2nd *et al.* ROR1 can interact with TCL1 and enhance leukemogenesis in Emu-TCL1 transgenic mice. *Proc Natl Acad Sci U S A* **111**, 793-798, doi:10.1073/pnas.1308374111 (2014).

2 Bichi, R. *et al.* Human chronic lymphocytic leukemia modeled in mouse by targeted TCL1 expression. *Proc Natl Acad Sci U S A* **99**, 6955-6960, doi:10.1073/pnas.102181599 (2002).
