# Supplementary material for: Wnt5a induces ROR1 dependent NF-κB activation to enhance MMP-9 expression and invasiveness in chronic lymphocytic leukemia
Source: Leukemia. 2025 Apr 28;39(7):1661–9. doi: 10.1038/s41375-025-02616-4 (PMC12208867; doi:10.1038/s41375-025-02616-4)

Supplementary Table 1. Biological features of 10 ROR1Neg and 12 ROR1Pos cases.

ID	ROR1 abMFI	IGHV	ZAP70	P53 status	Rai Stage
1	5.47	Unmutated	Unknown	Unknown	Stage II
2	4.86	Mutated	Negative	Unknown	Stage 0
3	6.55	Mutated	Negative	Unknown	Stage I
4	4.6	Mutated	Positive	Unknown	Stage II
5	4.4	Unknown	Positive	Unknown	Stage 0
6	0.66	Mutated	Negative	Wild type	Stage II
7	2.84	Mutated	Negative	Wild type	Stage II
8	3.0	Unmutated	Positive	Unknown	Stage I
9	3.54	Mutated	Negative	Wild type	Stage I
10	0.66	Mutated	Negative	Wild type	Stage II
11	54.06	Unmutated	Positive	Wild type	Stage IV
12	68.68	Mutated	Negative	Wild type	Stage II
13	65.83	Unmutated	Positive	Unknown	Stage III
14	73.95	Unmutated	Positive	Unknown	Stage IV
15	44.06	Mutated	Negative	Unknown	Stage III
16	55.2	Unmutated	Positive	Wild type	Stage I
17	52.17	Unmutated	Positive	Unknown	Stage II
18	49.35	Unmutated	Positive	Wild type	Stage IV
19	67.31	Unmutated	Positive	Unknown	Stage IV
20	100.9	Mutated	Negative	Unknown	Stage II
21	71.55	Unmutated	Positive	Wild type	Stage IV
22	92.72	Unmutated	Positive	Unknown	Stage IV

Supplementary Figure S1

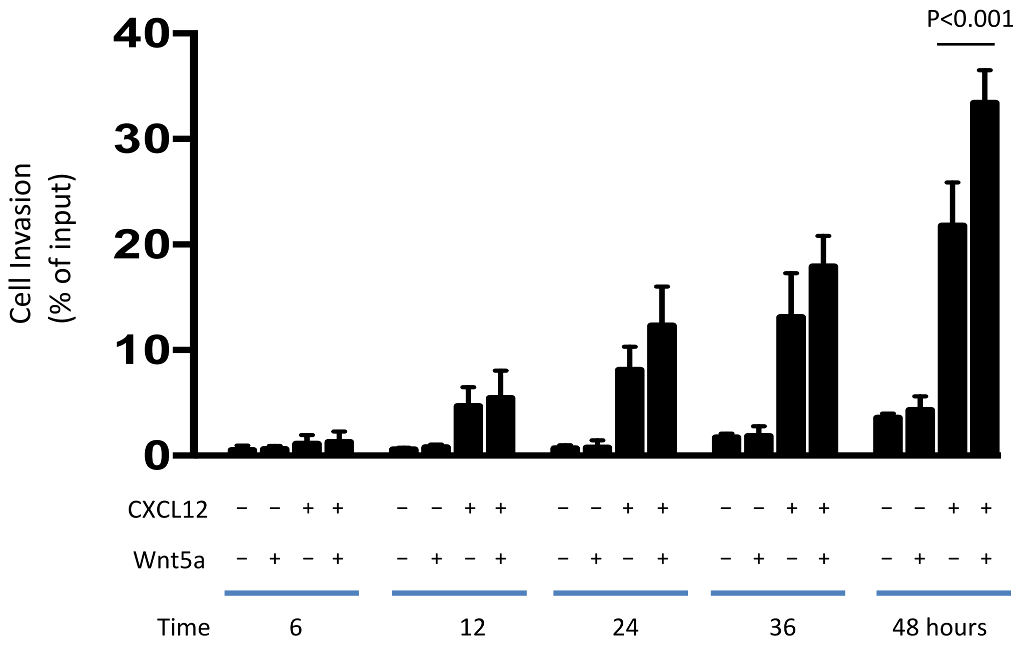

Supplementary Figure S2

A

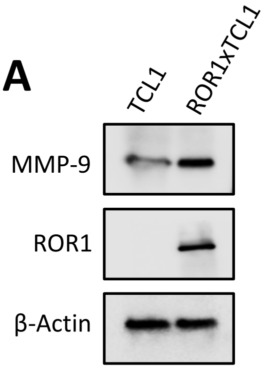

B

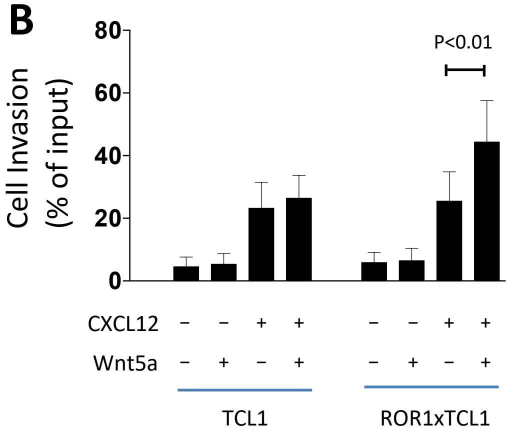

Supplementary Figure S3

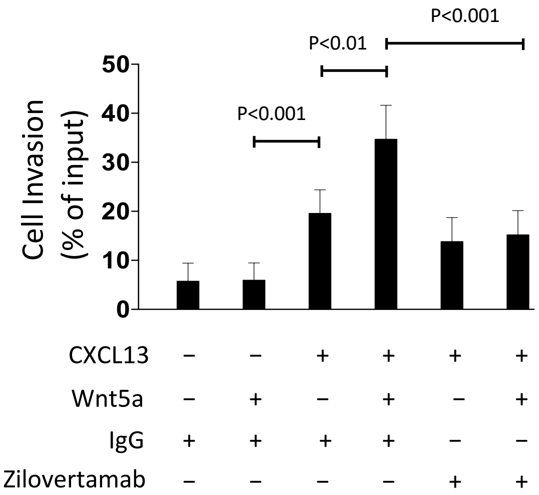

Supplementary Figure S4

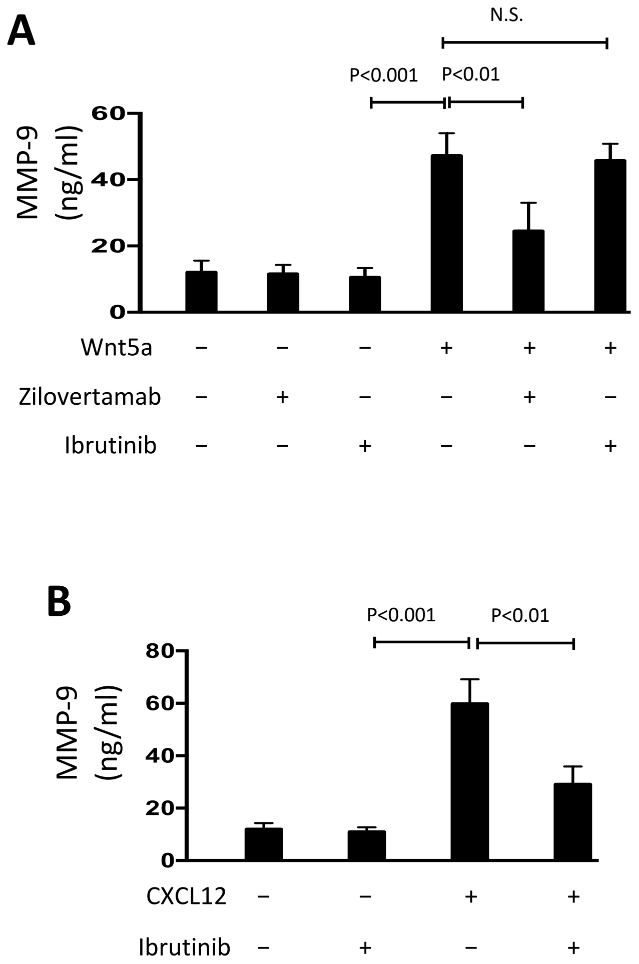

Supplementary Figure S5

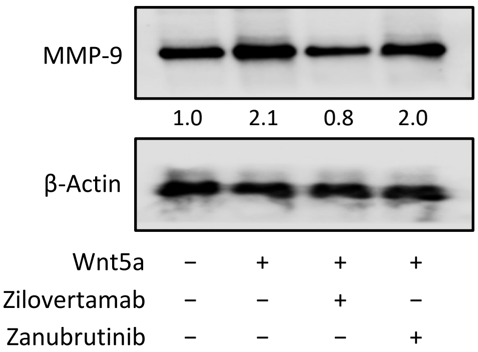

Supplementary Figure S6

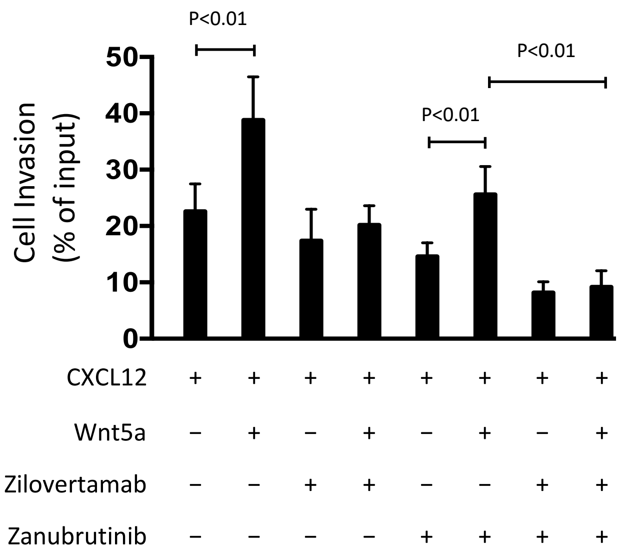

Supplement: Supplementary file 2 — Supplementary Figures [file 41375_2025_2616_MOESM2_ESM.pdf]
